# Expanded Beads of High Melt Strength Polypropylene Moldable at Low Steam Pressure by Foam Extrusion

**DOI:** 10.3390/polym14010205

**Published:** 2022-01-05

**Authors:** Daniele Tammaro, Alberto Ballesteros, Claudio Walker, Norbert Reichelt, Ulla Trommsdorff

**Affiliations:** 1 Department of Chemical, Materials and Production Engineering, University of Naples Federico II, P.le Tecchio 80, 8008 Napoli, Italy; 2 Sulzer Chemtech, Else-Züblinstrasse 11, 8404 Winterthur, Switzerland; alberto.ballesteros@sulzer.com (A.B.); claudio.walker@sulzer.com (C.W.); ulla.trommsdorff@sulzer.com (U.T.); 3 Borealis Polyolefine GmbH, St.-Peter-Strasse 25, 4021 Linz, Austria; norbert.reichelt@borealisgroup.com

**Keywords:** bead foam, extrusion, foam, polypropylene, isobutane, CO_2_, density

## Abstract

We explore the foam extrusion of expanded polypropylene with a long chain branched random co-polypropylene to make its production process simpler and cheaper. The results show that the presence of long chain branches infer high melt strength and, hence, a wide foamability window. We explored the entire window of foaming conditions (namely, temperature and pressure) by means of an ad-hoc extrusion pilot line design. It is shown that the density of the beads can be varied from 20 to 100 kg/m^3^ using CO_2_ and isobutane as a blowing agent. The foamed beads were molded by steam-chest molding using moderate steam pressures of 0.3 to 0.35 MPa independently of the closed cell content. A characterization of the mechanical properties was performed on the molded parts. The steam molding pressure for sintering expanded polypropylene beads with a long chain branched random co-polypropylene is lower than the one usually needed for standard polypropylene beads by extrusion. The energy saving for the sintering makes the entire manufacturing processes cost efficient and can trigger new applications.

## 1. Introduction

Polymer bead foams are produced by a sintering process (usually, steam chest molding) of foamed polymer beads. The sintering process allows us to obtain complex shapes with low density, a unique feature among all the technologies to produce polymer foams [1]. For this reason, polymer bead foams have attracted enormous attention from various foam industries, and they are used in automotive, leisure, sport, and long-life packaging. In the early 1980s, expanded beads of polypropylene (ePP) were introduced into the market and, since then, their applications have been constantly growing [2]. In recent years, ePP applications in the automotive sector have been spreading rapidly (i.e., bumpers, exterior and interior energy absorbers, door panels, knee protection, tool kits, trunk liners, console components, seat backs, bolsters etc.) with the goal to minimize car weight and increase safety. Moreover, the current green revolution asks for more sustainable polymers, and polypropylene (PP) could be a good candidate to replace polystyrene (PS) because it has a more advantageous Life Cycle Assessment for many categories (e.g., Ozone depletion, Climate change, Water resource depletion etc.) [2].

One of the limitations to ePP application is the low production productivity and the higher price compared to expandable and expanded polystyrene beads (EPS). ePP can be produced with two different technologies; autoclave (it can be considered as the conventional technology) or foam extrusion [3]. The extrusion technology, compared to the autoclave, is more cost-efficient and assures higher productivity and more flexibility. Indeed, it allows the economical production of small batches of foamed beads because solubilization, compounding and expansion happens in a one-step process [3]. Moreover, the extrusion technology allows us to obtain a narrower distribution of foamed bead size and smaller particles than the autoclave case. However, it is challenging to achieve a proper foam structure and density [4]. This is the reason more than 90% of ePP on the market is produced by autoclave technology [5].

There has been extensive research on the foaming process of ePP by autoclave [6] and many patents can be found [7,8,9]; however, even though the literature recognizes the great potential of developing a foam extrusion technology for ePP for the earlier mentioned advantages, the foamability of ePP by extrusion is not investigated deeply and only few patents [10] present promising technology. It is believed that, despite the numerous advantages for using the extrusion technology, the resulting extruded ePP beads have poorer mechanical properties compared to the autoclave ones and require more energy for being sintered. However, no studies can be found in the scientific literature and no explanation of the governing phenomena was given.

The foaming of beads by foam extrusion is different from the autoclave foaming. In extrusion foaming, the polypropylene/gas solution passes through small channels (where it is subjected to a high pressure drop rate (PDR), usually much higher than the one experienced in autoclave foaming [11]) and the beads are cut under water by a fast knife that may affect the morphology. The cooling history is usually much faster in extrusion foaming than in autoclave foaming because of the turbulent water flow around the extruded beads [12]. The extruded beads that are cooled quickly crystallize within a strong temperature inhomogeneity across the cross section of the beads that could result in an inhomogeneous crystal structure and lower crystallinity content compared to the autoclave foamed beads [13]. The degree of crystallinity and/or shape of crystals have a strong effect on final mechanical properties in the foamed beads. Moreover, the autoclave technology allows us to obtain a double peak of melting [14] in ePP that simplifies the sintering of beads, which can be done at a steam pressure of 2–3 bar [15,16]. The extruded beads currently available on the market have a difficult molding process that requires more energy for welding the surfaces [17].

Herein, we present a study on producing expanded beads of random copolymer PP using a commercially available extrusion line. We present insights into the process and the characterization of the foamed PP beads produced by extrusion using isobutane and CO_2_ as blowing agent. The choice of the blowing agent has to comply with not only technical (solubility, diffusivity, boiling point etc.) and economic requirements, but environmental and safety requirements as well. Based on the previous considerations, we selected: (I) isobutane that is a good solvent for PP and its solubility is very high offering the possibility to achieve low density foams [18,19]; (II) CO_2_ has a lower solubility in PP than isobutane, but it is an environmentally friendly choice and it is also more economical than the isobutane. 

The resulting beads are characterized in terms of density and open cell content. Furthermore, steam chest molding was performed to investigate the molding and mechanical properties of sintered boards.

## 2. Materials and Methods

High melt strength polypropylene (HMS PP) was supplied by Borealis and it was used as received. The material exhibits strain hardening at high shear rates comparable with those one in a foaming experiment. The presence of strain hardening ensures a setting mechanism for the growing bubbles that avoids the collapse and the rupture of cell walls. Indeed, it is well known in the literature [20] that a high degree of close cells is required for optimizing the mechanical properties.

Moreover, two types of additives were tested: anti-shrinking agent (influencing the permeability of the blowing agent through the cell walls) and a nucleating agent masterbatch of talc microparticles (with a maximum size of 20 microns). The materials were extruded as received. The isobutene and CO_2_ at a purity of 99.8% were supplied by Linde (Switzerland). The materials used for the experimental campaigns were: (a) RACO PP name WB260HMS from Borealis, Linz, Austria; (b) anti-shrinking agent name Atmer 7300 from Croda, Munich, Germany; (c) nucleating agent name PEA0025793 from Croda, Germany.

The cell structure was analyzed by Scanning Electron Microscope (i.e., Hitachi TM 3000 SEM) on the samples cut in nitrogen. ImageJ software was used for shape identification. Density was measured by two methods: (a) multiple beads density: weighting 1 L of beads; and (b) single bead density done by ASTM D792 that is not affected by the beads shape. 

The open cell content was measured by pycnometer (i.e., Ultrapyc 5000 foam, Anton Paar, Munich, Germany) following the ASTM method D6226 using nitrogen. 

The average beads size and their sphericity was analyzed by the optical method using a Camsizer (model 0208, Retsch Technology, Geneve, Switzerland). The steam chest molding machine used for sintering the beads was a commercial molding machine Teubert TransTec72/52 PP (Teubert Maschinenbau GmbH, Blumberg, Germany).

Mechanical properties were measured according to ISO 844 for the compression test and DIN 1789 for the tensile tests, using a Zwick Z1485 testing machine (Zwick. GmbH & Co. KG, Ulm, Germany) with a displacement of 500 mm/min for the tensile strength test and a Zwick Z050 testing machine (Zwick. GmbH & Co. KG, Ulm, Germany) with a displacement of 5 mm/min for the compression test. The test specimens have parallel top and bottom surfaces and essentially perpendicular sides; the dimensions were 50 by 50 by 25 mm.

The linear rheological response was measured by means of a Physica MCR 301 Rheometer from Anton Paar. The measurements were performed under nitrogen atmosphere using a plate–plate system with a radius of 25 mm (PP25) under oscillatory conditions; the gap between the plates was set to 1 mm. The test sweeps the angular frequency from ω = 0.1 to 50 rad s^−1^, with a fixed strain rate of 1%. The non-linear rheological response of the HMS PP, at high shear rates and deformation, was measured by means of a stress-controlled microcapillary rheometer [21]. The microcapillary has a diameter of D = 70 μm and a land length of L = 1500 μm. Above the microcapillary there is a reservoir with diameter Dr = 1 mm and a converging section to facilitate sample loading. Nitrogen was used to impose the pressure and a camera used for observing the polymer strand at the capillary exit.

The results of density and molding were averaged over at least three samples.

### 2.1. Sulzer Extrusion Foaming Equipment and Process

**Figure 1:**
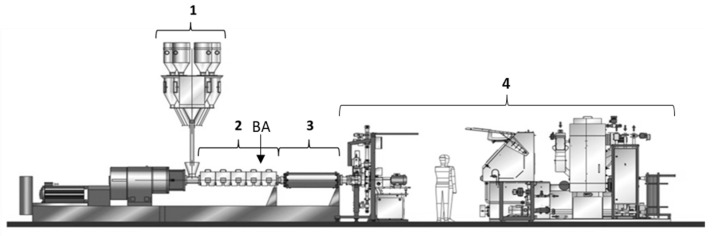
Sketch of the Sulzer extruder based ePP process as used in the trial (the person is depicted to give a size reference).

The expanded PP-beads were produced using the foam extrusion line from Sulzer (Winterthur, Switzerland, www.sulzer.com, accessed on 15 December 2021) optimized for ePP foams. Sulzer’s ePP foaming process consists of a combination of dosing devices, an inter-meshing co-rotating twin screw extruder, a melt conditioning section including melt cooler, melt pump and die plate (Figure 1). Sulzer operates a multi-purpose demonstration unit with ~20 kg/h capacity in its customer test center in Winterthur, Switzerland. 

Depending on the application, polymer granulate—virgin and/or recycled—can be used as the main feedstock (i.e., the part 1 in the Figure 1). Solid feed materials and additives are dosed separately by gravimetric feeders into the twin screw extruder (part 2 in Figure 1); the dispersion of the additives in the melt is controlled by the screw design and various operating parameters. The blowing agent is injected directly into the extruder via an injection valve (the exact position is pointed out by the arrow in Figure 1) and, under those conditions, the supercritical state is reached for CO_2_ [22]. The extruder has heating elements and water cooling, to maintain a constant and even temperature distribution throughout the housings and the melt.

The impregnated melt is transferred into a conditioning section mainly consisting of a heat exchanger (part 3 of Figure 1) and a melt pump. The heat-exchanger allows us to precisely set the temperature of the melt for optimal foaming conditions. The melt pump provides a constant flow rate of the melt into the underwater granulation system (part 4 in Figure 1). The person is depicted to give a size reference respect to the machine size, additional details are given in [23]. The temperature of the melt cooler and of the die is controlled by two independent thermal oil heat transfer units (HTU) and melt bolt thermocouples to measure the polymer processing temperature inside the polymer stream. The most relevant processing conditions are given in the results section, that are, the melt temperature at the foaming and pressure in the die plate.

### 2.2. Testing Procedure 

The foaming window was mapped for each formulation by investigating the effect of the foaming temperature and blowing agent concentration. The extruder temperature profile was kept constant to melt the PP properly. The melt cooler temperature was varied, and beads were collected when stable conditions were reached. The melt temperature was measured immediately before the die plate with a Gneuss (Bad Oeynhausen, Germany) temperature sensor equipped with a ceramic insulation close to the measuring tip (i.e., TF-CX-12A-00S-S0-F0-T1J-2G).

## 3. Results

**Figure 2:**
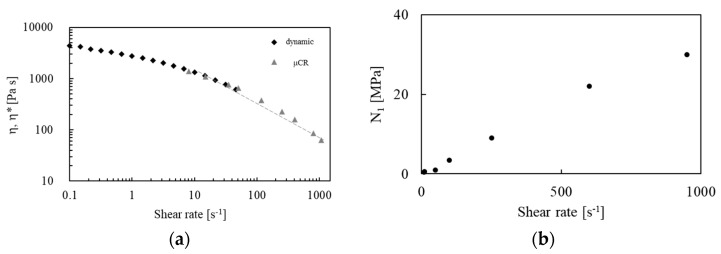
(**a**) Complex and shear viscosity as function of shear rate measured by dynamic (black rhombus) and capillary rheometer (grey triangles), the dashed line is the power law fitting; (**b**) normal stresses (N_1_) as function of shear rate.

A complete rheological characterization of the polymer is fundamental for designing a stable extrusion foam process of expanded PP beads. The linear and non-linear rheological behavior of HMS PP was investigated at 170 °C. The complex viscosity, η*, as a function of shear rates, is shown in Figure 2a (black rhombus); at low shear rates the viscosity approaches at a plateau value of ca. 5000 Pa s. The shear viscosity obtained by the microcapillary rheometer [20] is plotted in Figure 2a (grey triangles). The viscosity decreases dramatically at high shear rates, and it was modelled by fitting a power law equation (i.e., $\eta =K{\dot{\gamma }}^{n-1}$, where $\eta ,K,\;\dot{\gamma },\;n$ are the shear viscosity, the consistency, the shear rate and the flow index) from 20 to 1000 s^−1^. The fitting results have a coefficient of determination equal to 0.8 and show a flow index equal to 0.2, typical of a highly entangled polymer [24]. The observation of the exiting strands supplies important insights into the non-linear rheological behavior (e.g., die swell) that are directly linked to the extrusion of the material through the die plate for bead production. In particular, the observed die swell is used to calculate the first normal stresses difference, *N*_1_, (shown in Figure 2b), using the empirical equations [25,26,27]: $N_{1}^{2}=8\tau_{w}^{2}({\left(B-0.13\right)}^{6}-1),$ where $\tau_{w}^{}\;and\;B$ are the shear stresses at the wall and the die swell is defined as the ratio between the diameter of the polymer strand and the capillary diameter. It is worth noting that the swell measurements, *B*, can be considered independent of the geometry because the capillary design is characterized by *Dr/D* < 20 and *L/D* = 21 (where *Dr* is the diameter of the cylindrical reservoir) [27]. Moreover, the direct observation of the polymer flow provides qualitative information on the final foamed beads’ shape and size. The absence of melt fracture, in the entire range of shear rate investigated, makes this polymer particularly suitable for extrusion where a perfect control of the strand dimension is required.

### 3.1. Bead Properties

**Figure 3:**
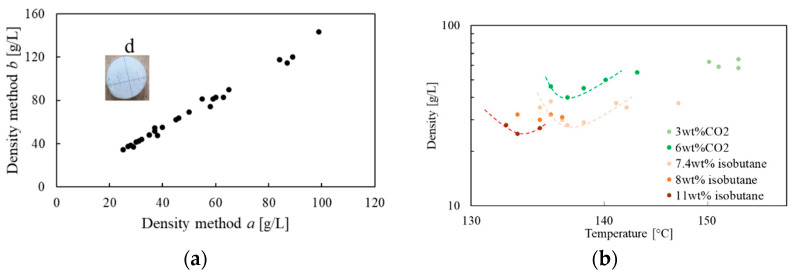
(**a**) Comparison between the density calculated with two methods; (**b**) density as function of foaming temperature. The dashed lines are neither a model nor a regression, they are guideline for eyes. They should help the reader identify the local minimum despite the scattered points.

The branched structure of random copolymer PP molecules exhibits rheological properties, such as high melt strength and rapid crystallization, that assure easy stabilization of the foaming beads. We tested WB260 HMS with isobutane and CO_2_ as blowing agents and compared the resulting foamed beads. The PP can be foamed within a broad range of melt temperature and density can be tuned between 100 to 20 g/L. The most relevant pressure for the foaming, the one at the die plate, was kept constant as much as possible between 150 and 200 bar. Figure 3a shows the comparison between the foam density measured with the two methods, *a* and *b*. As expected, method *a* (multiple beads density) always gives a lower density than the one measured with method *b* (single bead density) and the difference is ca. 40%. The insert *d* in Figure 3a shows the picture of an ePP bead with a foam density of 30 g/L. In the rest of the paper, density will always be the multiple beads density, that is, the density used by industry to define the requirements of ePP for automotives. Figure 3b shows the bulk density (in scale log-log) as a function of melt temperature, *T_foam_*, for five different blowing agent concentrations. For each concentration of blowing agent, we identify an optimum for foaming temperature, $T_{foam}^{optimal}$, at which we reach a low density and high concentration of a closed cell. *T_foam_* is a very important process variable for defining the final foam structure both in terms of density and morphology. A bell-shaped curve typically describes the effect of *T_foam_* on the foam density, with a maximum of the expansion ratio at optimal temperature, $T_{foam}^{optimal}.$ Below $T_{foam}^{optimal}$, the viscosity of the polymer increases, and crystallization or vitrification may occur [28,29,30]. Above $T_{foam}^{optimal}$, the reduction of the polymer viscosity induces bubble coalescence, and the increased diffusivity of the blowing agent is responsible for its loss through the external surface of the strand, followed by a corresponding reduction of foaming efficiency. All those phenomena hinder bubble growth. The foaming window is ca. 10 °C wide.

**Figure 4:**
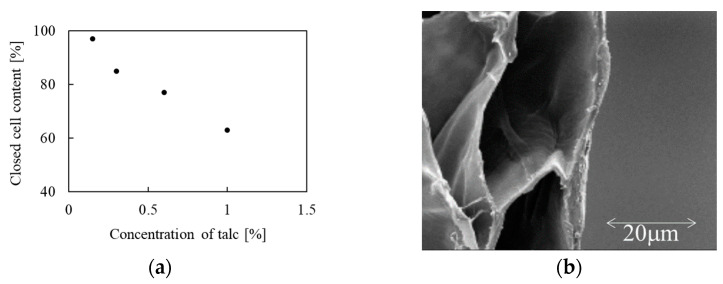
(**a**) Closed cell content as function of talc content; (**b**) cell wall thickness detail.

The effect of an anti-shrinking agent (i.e., GMS) and a nucleating agent (i.e., talc) on the cell morphology of ePP foamed with 7.4 wt% of isobutane was investigated at the optimum foaming temperatures. Figure 4a shows a rapid decrease of closed cell content when the talc concentration is increased. In our case, the talc particle size of 20 microns is much bigger than the thickness of the cell walls (Figure 4b) and we can speculate that during the bubble growth the accumulation of stresses around rigid solid talc particles leads to the breaking of cell walls. On the other hand, we did not observe any sensitive changes in open cell content as a function of GMS concentration that acts as a lubricating agent.

### 3.2. Molding

Five sets of extruded beads were molded into the shape of boards later used for mechanical property determination: low, medium, and high-density beads produced using isobutane as the blowing agent (Sulzer 1, 2 and 3) and low and medium density beads produced using CO_2_ as the blowing agent (Sulzer 4 and 5). The molding of extruded beads was achieved using steam pressure to 3 bar (the details are given in Table 1). All the samples were well sintered, having straight surfaces without wrinkles and shrinking of the object. The increase of density after the steam chest molding process is shown in Table 1. When formulations were submitted to the molding process the final part densities of the materials differ around 40 g/L compared to the bulk density values. The increment perceived was related to the pressure filling used (higher filling pressures produce molds with bigger final part densities) due to the compaction of the beads during the molding process.

### 3.3. Mechanical Properties

The boards molded as described above were analyzed for their mechanical properties. In Table 2 and Table 3, mechanical properties are displayed along with the respective density values of the measured samples. The tensile strength and elongation at break after the steam chest molding of the board (i.e., thickness 20 mm) are shown in Table 2.

The compression test values at 25%, 50% and 75% of compressive strain after the steam chest molding of the board (i.e., thickness 60 mm) are shown in Table 3.

No direct comparison between the mechanical properties of boards produced with the described extruded beads and those produced with beads of the same material but produced with the autoclave process, is available. However, compared to commercially available ePP-beads produced with the autoclave method, the described beads reach somewhat lower specific mechanical properties [31,32]. Autoclave foaming technology allows PP to foam at temperatures comparably close to its melting temperatures, which is not possible in an extrusion process due to viscosity. The rearrangement of crystalline structure in PP in impregnation and foaming steps during the autoclave process leads to the development of a double melting peak in the expanded beads and higher mechanical properties caused by the presence of two different crystalline phase structures combined with higher crystallinity [33]. This very characteristic annealing effect of ePP beads made by the autoclave process is not observed in beads produced by the extrusion bead process.

The tensile and mechanical compression properties of boards (Sulzer 1–5) are linearly dependent on the final part density of the piece. Furthermore, it was found that there is no significant difference between the mechanical properties of the beads produced with isobutane compared to the ones foamed with carbon dioxide.

## 4. Conclusions

The foamability of random co-polypropylene by foam extrusion of expanded beads was explored. The results show that all polypropylene grades can be foamed with a fine tuning of processing conditions and the densities can be varied from 20 to 100 kg/m^3^. The optimal foaming temperature was investigated by changing the melt temperature and concentrations of isobutene and CO_2_. The curve of density as a function of temperature has the classical bell shape and the minimum ranges from 140 to 130 °C depending on the gas concentration. In particular, it decreases with the concentration of the blowing agent. The steam chest molding of the foamed beads shows that it is possible to sinter the beads in a wide range of steam pressures (from 0.25 to 0.3 MPa) and a cycle time from 110 to 228 s. The compression test on the molded parts shows that the compression strength at 25%, 50% and 75% (i.e., from 200 to 800 kPa) is slightly lower than for the ePP by autoclave reported in the literature. Surprisingly, the sintering of beads can be performed independently on the closed cell content and at only 3 bar steam pressure. The energy saving for the sintering makes the entire manufacturing process cost efficient and can trigger new applications.

## Figures and Tables

**Table 1:** Steam chest molding conditions and density of the molded parts.

Property	Sulzer 1	Sulzer 2	Sulzer 3	Sulzer 4	Sulzer 5
Blowing agent	isobutane	isobutane	isobutane	CO_2_	CO_2_
Bulk Density of beads [kg/m^3^]	26 ± 2	43 ± 2	103 ± 1	35 ± 1	58 ± 1
Open cell content [%]	20 ± 1	8 ± 1	1 ± 1	34 ± 1	26 ± 1
Moulding pressure [MPa] (28 mm plate)	0.3 ± 0.01	0.3 ± 0.01	0.3 ± 0.01	0.28 ± 0.01	0.28 ± 0.01
Moulding cycle time [s]	228 ± 1	228 ± 1	159 ± 1	118 ± 1	110 ± 1

**Table 2:** Mechanical properties of molded ePP.

Property	Sulzer 1	Sulzer 2	Sulzer 3	Sulzer 4	Sulzer 5
Blowing agent	isobutane	isobutane	isobutane	CO_2_	CO_2_
Density of test piece [kg/m^3^]	60 ± 1	83 ± 1	137 ± 6	52 ± 2	76 ± 2
Tensile strength [kPa]	340 ± 6	441 ± 22	589 ± 15	266 ± 11	411 ± 24
Elongation at break [%]	20 ± 1	15 ± 3	12 ± 1	12.8 ± 2	11.3 ± 1

**Table 3:** Compression test mechanical properties of the materials.

Property	Sulzer 1	Sulzer 2	Sulzer 3	Sulzer 4	Sulzer 5
Blowing agent	isobutane	isobutane	isobutane	CO_2_	CO_2_
Density of test piece [kg/m^3^]	65 ± 2	90 ± 2	150 ± 3	51 ± 1	75 ± 2
Compression strength 25% [kPa]	139 ± 4	228 ± 3	529 ± 33	128 ± 2	252 ± 15
Compression strength 50% [kPa]	207 ± 2	339 ± 4	813 ± 63	196 ± 4	405 ± 22
Compression strength 75% [kPa]	405 ± 7	715 ± 14	2037 ± 252	456 ± 12	1061 ± 62

## Data Availability

Data is contained within the article.
